# Oxytocin vs. placebo effects on intrusive memory consolidation using a trauma film paradigm: a randomized, controlled experimental study in healthy women

**DOI:** 10.1038/s41398-023-02339-z

**Published:** 2023-02-04

**Authors:** Tolou Maslahati, Katja Wingenfeld, Julian Hellmann-Regen, Julia Kraft, Jing Lyu, Marie Keinert, Aline Voß, An Bin Cho, Stephan Ripke, Christian Otte, Katharina Schultebraucks, Stefan Roepke

**Affiliations:** 1 Department of Psychiatry and Psychotherapy, CBF, Charité – Universitätsmedizin Berlin, corporate member of Freie Universität Berlin, Humboldt-Universität zu Berlin, and Berlin Institute of Health, Berlin, Germany; 2 Department of Psychiatry and Psychotherapy, CCM, Charité – Universitätsmedizin Berlin, corporate member of Freie Universität Berlin, Humboldt-Universität zu Berlin, and Berlin Institute of Health, Berlin, Germany; 3 Department of Biostatistics, Columbia University, Mailman School of Public Health, New York, NY USA; 4 Department of Clinical Psychology and Psychotherapy, Friedrich-Alexander-Universität Erlangen-Nürnberg, Erlangen, Germany; 5 Stanley Center for Psychiatric Research, Broad Institute of MIT and Harvard, Cambridge, MA USA; 6 Analytic and Translational Genetics Unit, Massachusetts General Hospital, Boston, MA USA; 7 Department of Psychiatry, NYU Grossman School of Medicine, New York, NY USA; 8 Department of Population Health, NYU Grossman School of Medicine, New York, NY USA

**Keywords:** Learning and memory, Predictive markers, Psychiatric disorders, Psychology

## Abstract

Oxytocin administration during a trauma analogue has been shown to increase intrusive memories, which are a core symptom of post-traumatic stress disorder (PTSD). However, it is unknown whether oxytocin influences the acquisition or the consolidation of the trauma. The current study investigates the effect of the activation of the oxytocin system during the consolidation of an analogue trauma on the formation of intrusive memories over four consecutive days and whether this effect is influenced by individual neurobiological, genetic, or psychological factors. We conducted a randomized double-blind placebo-controlled study in 217 healthy women. They received either a single dose of intranasal oxytocin (24 IU) or placebo after exposure to a trauma film paradigm, which reliably induces intrusive memories. We used a general random forest to examine a potential heterogeneous treatment effect of oxytocin on the consolidation of intrusive memories. Furthermore, we used a poisson regression to examine whether salivary alpha amylase activity (sAA) as a marker of noradrenergic activity and cortisol response to the film, polygenic risk score (PRS) for psychiatric disorders, and psychological factors influence the number of intrusive memories. We found no significant effect of oxytocin on the formation of intrusive memories (*F*(2, 543.16) = 0.75, *p* = 0.51, *ηp2* = 0.00) and identified no heterogeneous treatment effect. We replicated previous associations of the PRS for PTSD, sAA and the cortisol response on intrusive memories. We further found a positive association between high trait anxiety and intrusive memories, and a negative association between the emotion regulation strategy reappraisal and intrusive memories. Data of the present study suggest that the consolidation of intrusive memories in women is modulated by genetic, neurobiological and psychological factors, but is not influenced by oxytocin. Trial registration: NCT03875391.

## Introduction

Post-traumatic stress disorder (PTSD) is a psychiatric disorder with high individual and societal burden [1]. Individuals with PTSD are at risk of a chronic course of the disorder [2, 3] and often face physical and psychiatric comorbidities [2], as well as social and occupational disabilities [4]. Exposure to a traumatic event is a mandatory criterion for a PTSD diagnosis [4]. Nevertheless, only a minority of those exposed to traumata develop a PTSD [5]. It remains a crucial challenge to identify vulnerability factors of PTSD. Results regarding the association of PTSD and hormonal and neuroendocrine dysregulation such as the hypothalamic-pituitary-adrenal (HPA) axis and the noradrenergic system are heterogeneous [6, 7]. Biomarkers that have been shown to influence the development of PTSD are cortisol, salivary α-amylase (sAA), and heart rate variability (HRV) [8–12]. Furthermore, common genetic variants have been identified in Genome-Wide Association Studies (GWAS) as risk factors for PTSD [13, 14]. Findings from GWAS can be utilized to compose polygenic risk scores (PRS) that capture the genetic propensity of an individual towards a given trait or disorders in independent samples [15]. As such, PRS have provided a useful approach to investigate the joint effect of many genetic risk variants on disease risk and other phenotypes [14]. The PTSD PRS has been shown to be associated with the onset and severity of PTSD [13, 14]. Furthermore, different psychiatric disorders have been shown to share common genetic risk variants to varying degrees [16–18]. Cross-disorder GWAS also enable us to derive PRS that reflect genetic liability to psychopathology and explore their effects on symptom dimensions. Recent genome-wide findings also highlight genetic correlations between PTSD and other psychiatric disorders, such as Major Depressive Disorder (MDD) and Schizophrenia (SCZ), as well PTSD symptoms [19]. Therefore, it is is likely that PRS for other disorders may be informative of the occurrence of PTSD symptoms.

Intrusive memories are a core symptom of PTSD and are defined by recurrent involuntary distressing recollections or nightmares of the experienced trauma [4]. Although intrusive memories after traumatic experiences are part of a nonpathological adaption process [20], their frequency [21, 22], their vividness, and their perceived distress are predictive of the development of PTSD [23–26]. The understanding of the development of intrusive memories is crucial for a better comprehension of PTSD and withholds a potential for administering interventions early post-trauma to prevent adverse outcomes.

The oxytocin system has been implicated to play a role in various psychiatric disorders [27], including PTSD [28–30]. According to the social salience hypothesis [31], oxytocin is thought to enhance the sensibility to salient cues in the surrounding [28, 32]. Oxytocin may facilitate adaption to high stress, such as traumatic events [33–36], by increasing the salience of safety signals in a positively interpreted environment. In contrast, it may also enhance the salience of negative cues, resulting in anxiety and elevated stress levels in a social context that is interpreted as unpredictable or threatening [31]. Consistent with the social salience hypothesis [31], intranasal oxytocin administration before a trauma film caused an increase in intrusive memories, in a previously conducted study [12]. However, these results do not allow conclusions on whether oxytocin impacted the acquisition or the consolidation of the trauma film. The time window, in which most of the consolidation is thought to occur, lasts six hours [37, 38]. As oxytocin was administered before the trauma film, its effect peaked during the film (acquisition phase) and endured until after the film had ended (consolidation phase) [39]. Therefore, it might also have influenced the critical time window of memory consolidation.

As the timing of oxytocin administration seems to play a crucial role in its effects, the current study examined the effect of oxytocin administration after a traumatic event on the consolidation of intrusive memories. Previous studies found that oxytocin administration acted anxiolytically after traumatic events [40–42] and reduced PTSD symptoms [40, 43, 44]. It further facilitated fear extinction and memory consolidation when administered after memory acquisition in rodent studies [45, 46] and in humans [47]. We therefore hypothesized a decreased frequency of intrusive memories after oxytocin administration compared with placebo over the following four days. To address the complexity of PTSD development, we included a variety of predictors in the analysis using Machine Learning (ML) approaches. All included predictors have previously been associated with PTSD risk and consisted of psychological, genetic, and neurobiological variables [8–10, 12, 13, 48]. ML approaches provide the possibility to investigate a variety of variables and their complex interactions [49]. Using ML approaches, it has been possible to identify biomarkers and multiple polygenic risk scores associated with posttraumatic stress [12, 50–52].

## Materials and methods

The current randomized, experimental, double-blind, placebo-controlled study was performed at the Department of Psychiatry and Neuroscience, Charité — Universitätsmedizin Berlin, Campus Benjamin Franklin. The local ethics committee of Charité —Universitätsmedizin Berlin approved the study protocol (EA4/162/18). Participants received all information at least 24 h in advance and signed written informed consent upon arrival in the laboratory. The start of every testing was set for 2 pm, to account for differences in cortisol levels due to the circadian rhythm into consideration [53]. Each testing lasted 125 min. Further test conditions have been described and published formerly [11].

### Participants

**Fig. 1:**
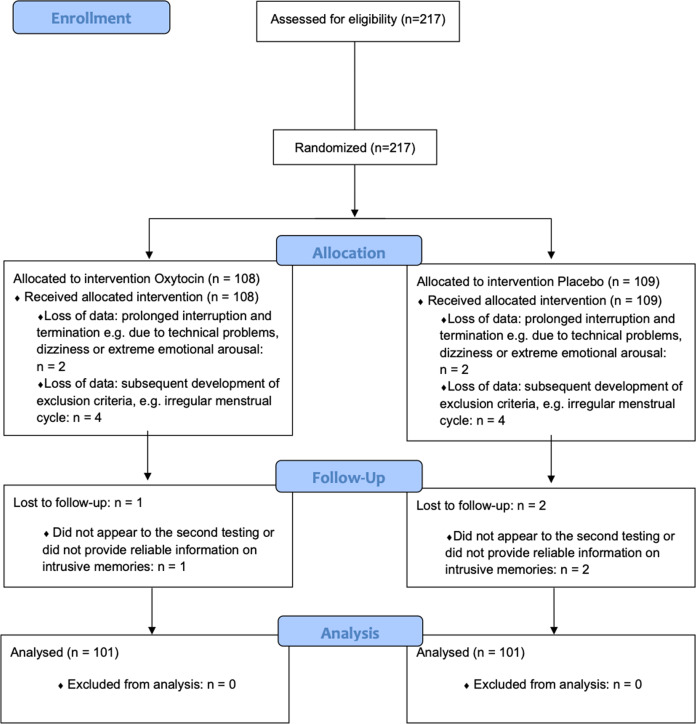
Flow diagram. Flow chart showing the inclusion and exclusion of participants during the course of the study.

217 healthy female participants were recruited via public postings or email lists (Fig. 1). As the oxytocin effect is sexually dimorphic [54], the study only encompassed female participants. Eligibility criteria, which were assessed before participation, included mental and physical health-related aspects and have been listed previously [11] and in the supplement information. As the menstrual cycle has been shown to have an impact on endogenous oxytocin levels [55] and intrusion formation [56, 57], participants, with no usage of hormonal contraception, were tested during their luteal phase only. Participants were asked about the first day of their last menstruation and the length of their cycle length. These data were used to calculate the phase of menstrual cycle for each participant. Pregnancy was ruled out, using HCG ULTRA pregnancy tests. All participants received an expense allowance of 50 €. To assure full recovery from the trauma film, participants were contacted via phone four weeks after the experiment and were offered psychological care, in case of any ongoing distress. One participant reported ongoing intrusive memories and received six counseling sessions with a licensed psychologist. The intrusive memories and associated distress disappeared in the course of aftercare for this participant. A priori power analysis indicated that a sample size of 200 would be needed to detect small to moderate effects (Cohen’s d = 0.4, α = 0.05, power = 0.8). To factor in possible dropouts, 217 participants were enrolled.

### Experimental phase

Participants were randomly assigned to the experimental (oxytocin) or the placebo group using Research Randomizer [58] and received either 24 IU oxytocin (nasal spray, Syntocinon®) or a placebo preparation (sodium chloride nasal spray) in accordance with their group allocation. Intranasal administration took place once after the trauma film. Both participant, and examiner were blind to the treatment condition. To ensure the double-blind design of the study both treatment sprays looked identical. The examiner was blinded during the measurement of the primary outcome and statistical analysis.

Alterations of cortisol levels, sAA activity, and HRV were assessed seven times during the study: at baseline, directly before the trauma film, and five times every 15 min after the trauma film. Participants received instructions on how to fill out the intrusion diary over the following four days. Psychometric assessment included Beck Depression Inventory-Revised (BDI-II), Childhood Trauma Questionnaire (CTQ), Emotion Regulation Questionnaire (ERQ) and State-Trait Anxiety Inventory - Trait (STAI - T). Further information about the questionnaires, the analog trauma paradigm, the intrusion diary, and the measurement of biomarkers (salivary assessment and HRV) are described in the Supplement Information and have been published previously [11]. Chronicle structure of the study procedure is depicted in the Supplementary Fig. 1.

### Genetic data processing

To generate genome-wide data, blood-derived DNA samples were assayed for > 700,000 Single Nucleotide Polymorphisms (SNPs) on the Illumina Global Screening Array BeadChip (Illumina, San Diego, CA). All subsequent analysis steps, which are described elsewhere in greater detail [59, 60], were carried out using standard software e.g. PLINK [61] and a previously established pipeline for analyzing SNP data [62]. Quality checks were performed to remove SNPs with high missingness (>2%), Hardy-Weinberg equilibrium violations (*P* < 10^−6^) and autosomal heterozygosity deviations (|F_het_ | > 0.2). Next, principal component analysis (PCA) and relatedness testing were conducted on a set of 92,252 autosomal SNPs obtained after linkage disequilibrium (LD) pruning (r^2^ > 0.02) and frequency filtering (MAF > 5%). Ancestry outlier identified by Principal Component Analysis (see Supplementary Fig. 2) as one individual of a related pairs were removed from the sample. (PI-HAT > 0.2). Missing genotypes were imputed using the publicly available HRC reference panel release 1.1 [57] resulting in a set of 9,059,416 SNPs.

PRS that index genetic liability towards MDD [58], SCZ [59], PTSD [14] and psychiatric disorders (“Cross-Disorder” [CD]; [16]) were calculated in the present sample. A clumping procedure was applied to respective GWAS summary statistics to determine SNPs that are independently associated with each trait. Quality-controlled SNPs (MAF > 5% and INFO scores >0.9 if available) were clumped using LD parameters of r^2^ ≥ 0.1 within a 500 kb window. PRS were then generated by multiplying GWAS-derived effect sizes (log OR) with imputed SNP dosages of the effect allele summed over each individual across the genome. This step was repeated to obtain individual-level and genome-wide PRS for all traits of interest, the number of SNPs used to construct each PRS can be found in the Supplementary Table 1. To account for remaining population stratification within the European sample the first four PCs were added as covariates in the final statistical model.

### Statistical analysis

We used Chi-square and Student´s *t*-test or the non-parametric Mann-Whitney-*U*-test to compare sample characteristics between the oxytocin and the placebo group. To test whether treatment blinding was successful, we performed binomial tests in both treatment groups.

### Group differences and heterogenous treatment effects of oxytocin

We conducted a repeated-measures mixed design ANOVA to assess the effect of oxytocin (between-subject factor) and time (within-subjects variable with four levels) on the number of intrusive memories. Homogeneity of variance was assessed by Levene’s statistic and sphericity was examined with Mauchly’s test. Greenhouse-Geisser corrected *p* values are reported, because the assumption of sphericity was not met. We further estimated the average treatment effects (ATEs) to examine mean group differences based on targeted maximum likelihood learning [62] of the generalized random forest (GRF) approach. If intervention effects (ATEs) are heterogenous within treatment groups and vary with differences in covariate space, heterogeneity in treatment effects per group, is not best represented by the mean. We therefore examined the heterogeneous treatment effects (HTE) to determine whether the ATE varied depending on differences in the covariate space, i.e., whether the ATE were nonhomogenous within the treatment group. We used GRF to run a non-parametric omnibus hypothesis test about the dimension of differences in treatment effects across subsets of the population [63]. We extended the classical random forest algorithm (74, 75) by using recursive portioning of the data. Based on prior literature, multiple biological (i.e., delta cortisol or sAA), genetic characteristics and psychological constructs (trait anxiety and emotional regulation strategies) were included as covariates to moderate the effect of oxytocin per group. The GRF [62] is a principled statistical method, which allows a data-driven way to systematically estimate heterogeneity in treatment effects [64]. To examine which variables, predict variance in the oxytocin effects best, we ranked the covariates in order of importance. A more detailed description of this approach has been published previously [12].

### Poisson regression—predictors influencing the number of intrusive memories

We further examined which covariates have a direct impact on intrusive memories, by applying a stepwise Poisson regression.

## Results

In the final analysis we included 202 out of the 217 participants that were enrolled and randomized to either oxytocin or the placebo condition. Inclusion and exclusion of participants during the study are depicted in Fig. 1. Regarding the characteristics presented in Table 1, the 202 analyzed participants did not significantly differ from the 15 participants that were excluded (Supplementary Table 2). There were also no significant differences regarding the characteristics presented in Table 1 between the oxytocin (*n* = 101) and the placebo group (*n* = 101).

**Table 1:** Sample characteristics.

Characteristics	Oxytocin (*n* = 101) *M* (*SD*) or *n*	Placebo (*n* = 101) *M* (*SD*) or *n*	Statistics
Age	25.08 (6.01)	25.19 (5.99)	*t*(200) = −0.13, *p* = .89, *d* = −0.02 (95% CI [−1.78, 1.56])
Intake of oral contraceptives	29	30	χ*2*(1) = 0.95, *p* = .88, φ = 0.01
Current Smoker	22	33	χ*2*(1) = 3.02, *p* = .08, φ = 0.12
BMI	22.04 (2.88)	21.99 (2.41)	*t*(200) = 0.09, *p* = .92, *d* = 0.02 (95% CI [−0.77, 0.77])
CTQ	31.42 (7.89)	32.62 (8.73)	*U* = 4551, Z = −1.33 *p* = .19
STAI-T	32.53 (6.62)	32.66 (6.56)	*U* = 4884, Z = −0.28 *p* = .77
BDI-II	4.26 (4.29)	4.07 (3.56)	*U* = 5027.50, *Z* = − 0.18 *p* = .86
ERQ reappraisal	28.81 (5.69)	30.17 (4.63)	*t*(200) = −1.85, *p* = .07 *d* = −0.26 (95% CI [−2.81, 0.08])
ERQ suppression	11.72 (4.46)	11.96 (4.24)	*t*(200) = −0.39, *p* = .69, *d* = −0.05 (95% CI [−1.83, 1.36])
Participant who had seen the film before	9	5	χ*2*(1) = 1.19, *p* = .28, φ = 0.08

In case the normality assumption for residuals was violated, the Mann–Whitney *U* test was used (*U*).

*M* mean, *SD* standard deviation, *BMI* body mass index, *CTQ* childhood trauma questionnaire, *STAI-T* state-trait anxiety inventory-trait subscale, *BDI-II* Beck depression inventory-revised, *ERQ* emotion regulation questionnaire (subscales reappraisal and suppression).

The longitudinal development of salivary cortisol, sAA activity, and HRV before and after the trauma film and before and after administration of oxytocin vs. placebo is illustrated in Supplementary Fig. 3. There was a significant increase in cortisol levels (*F*(2.18, 433.24) = 16.89, *p* ≤ .001), sAA activity (*F*(4.21, 842.60) = 6.16, *p* ≤ .001), and HRV (*F*(1.83, 353.07) = 4.93, *p* = .01) after the trauma film. There was no significant difference in cortisol levels (*F*(2.18, 433.24) = 0.76, *p* = .48), sAA activity (*F*(4.21, 842.60) = 0.91, *p* = .46), or HRV (*F*(1.83, 353.07) = 0.76, *p* = .46) between the oxytocin and placebo group.

Manipulation check showed that the film was perceived as a stressor by the participants: Participants were asked to rate how much distress they felt after watching the trauma film (1 = not at all to 5 = very distressed). Average distress-ratings (oxytocin: mean = 4.82 ± 1.06 *SD*, placebo: 4.76 ± 1.14 *SD*) of the trauma film exceeded the cut-off score of 3, defined by Weidmann et al. [65]. The two groups did not differ significantly in their ratings (*t*(200) = 0.36, *p* = .72, Cohen’s *d* = 0.05, 95% CI −0.25 to 0.36). The blinding of treatment condition was also successful: The binomial tests indicated that the proportion of correct guesses did not significantly differ from theoretical frequency (oxytocin: *p* = .31, placebo: *p* = .35). Proportion of correct guesses were 55.2% in the oxytocin group and 44.68% in the placebo group.

### Genetic data

A total of 206 blood-derived DNA samples were obtained from the participants and assayed on the Illumina Global Screening Array BeadChip (Illumina, San Diego, CA). Due to subsequent quality checks one individual was excluded due to a low call rate (<98%). Next, 29 ancestry outliers were removed after principal component analysis. Additionally, one individual of a genetically related pair (PI-HAT > 0.2) was removed at random. After exclusion, genotypes of 175 individuals were imputed using the publicly available HRC reference panel release 1.1 [22] and utilized for polygenic scoring.

### Group differences and average treatment effect

**Fig. 2:**
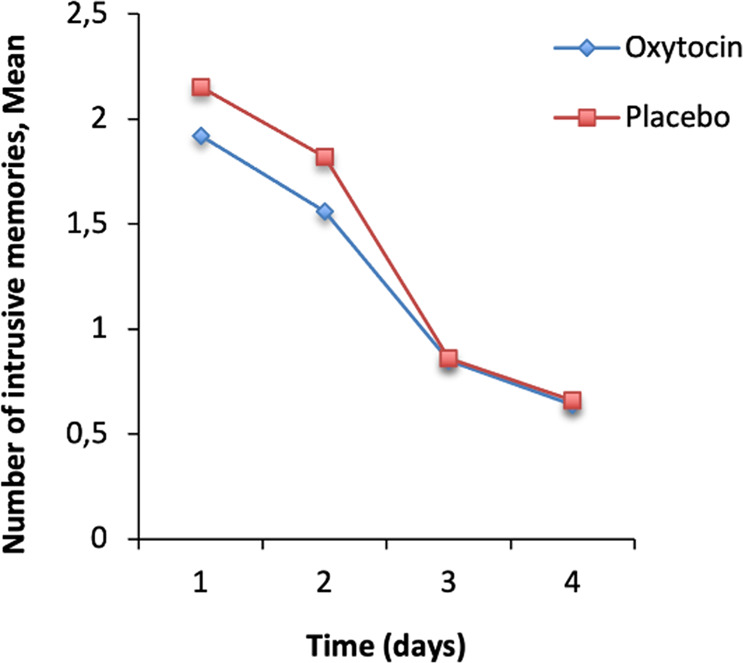
Mean number of intrusive memories in the oxytocin and placebo group over 4 days. Points are means.

The mean number of intrusive memories of both groups (oxytocin and placebo) over 4 days are shown in Fig. 2. While the number of intrusive memories expectedly declined over time (*F*(2.73, 543.16) = 74.83, *p* < .001, *η*_*p*_^*2*^ = 0.27), there was no significant treatment effect (*F*(2, 543.16) = 0.75, *p* = .51, *η*_*p*_^*2*^ = 0.00). We further found no significant interaction of time and treatment (*F*(1, 199) = 0.68, *p* = .41, *η*_*p*_^*2*^ = 0.00). The ATE was − 0.38 (*SE* = 0.64), i.e., the oxytocin group shows on average 0.38 less intrusions than the placebo group.

### Heterogenous treatment effect

To further investigate the heterogeneity of the treatment we used the omnibus test for the differential forest prediction (estimate = 0.23, *SE* = 0.61, *t* = −0.39, *p* = .35) indicating no heterogeneity in the intervention effect [64]. As estimated propensity scores were not close to one or zero, the overlap assumption was fulfilled (Supplementary Fig. 4). Supplementary Fig. 5 shows the importance ranking for variables predicting the conditional average treatment effect (CATE) of oxytocin administration on the number of intrusive memories. Variables ranking the highest were the total score of the CTQ [66], SCZ PRS, delta sAA, and ERQ - Reappraisal (-R).

### Poisson regression

**Fig. 3:**
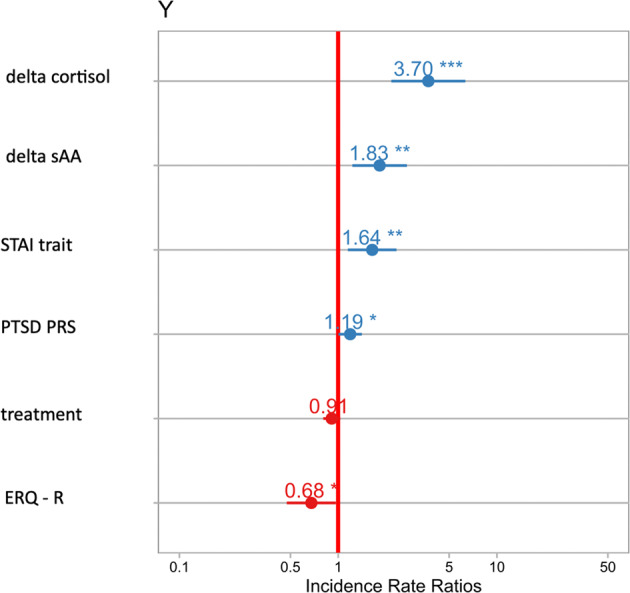
Coefficient estimates of the step-wise poisson regression (see Table 2). Significant effects are marked by an asterisks (* < 0.05, ** < 0.01, *** < 0.001). Highest coefficients are plotted on top, vertical red line indicates the intercept of “no effect”. STAI trait state-trait anxiety inventory-trait subscale, PTSD PRS = post-traumatic stress disorder polygenic risk score, ERQ-R = emotion regulation questionnaire – reappraisal subscale.

We fitted a Poisson regression using a stepwise approach to examine how those features predict the number of intrusive memories. We included the most important features from the CATE analysis (Fig. 3, i.e., CTQ, delta sAA, delta cortisol, STAI - T [67], ERQ-R [68], PTSD PRS, MDD PRS, SCZ PRS, Cross PRS plus the treatment (oxytocin vs. place)). PTSD PRS, STAI - T, delta sAA, delta cortisol, and ERQ-R remained significant predictors of the number of intrusive memories (Table 2 and Fig. 3). Hence, these variables had a direct effect on the number of intrusive memories.

**Table 2:** Stepwise Poisson regression.

	Estimate	Std. error	*z*-value	*p*-value
(Intercept)	0.96	0.19	5.17	2.40e^−05^***
Treatment (oxytocin vs. placebo)	−0.09	0.06	−1.48	0.14
Delta amylase	0.60	0.20	2.98	<0.001**
Delta cortisol	1.31	0.27	4.77	1.81e^−06^***
STAI-T	0.49	0.18	2.74	<0.001**
ERQ-R	−0.39	0.18	−2.14	0.03*
PTSD PRS	0.17	0.09	2.01	0.04*

*STAI-T* state-trait anxiety inventory-trait subscale, *ERQ-R* emotion regulation questionnaire – reappraisal subscale, *PTSD PRS* post- traumatic stress disorder polygenic risk score.

Significance thresholds: 0.00 = ***, 0.001 = **, 0.01 = *. Deviance residuals: Min. –5.15, 1Q = −1.55, Median = −0.52, 3Q = 1.07, Max. = 5.11, AIC: 1339.8.

## Discussion

In this study, we examined the effects of exogenous oxytocin administration vs. placebo in an experimental trauma film paradigm on the consolidation of intrusive memories. Previous data have shown enhancing effects of oxytocin on memory consolidation and fear extinction in rodents and in humans when administered after memory acquisition [45–47] and anxiolytic effects in PTSD patients after trauma [40–42]. We, therefore, hypothesized that a single intranasal dose of oxytocin at the time of consolidation of a stressful event (after watching the trauma film) leads to a decreased frequency of intrusive memories compared with placebo over the following four days. Contrary to our hypothesis, healthy women did not show significantly fewer intrusive memories after oxytocin over the four following days compared to the placebo group.

In contrast to the current results, we found a significantly increased number of intrusive memories when oxytocin was administered before an analogue trauma (before acquisition) in a previous study [12]. Accordingly, the effect of oxytocin administration on intrusions seems to be dependent on the phase of memory processing.

Considering the results of the current study and the previously published study [12], the findings allow us to disentangle, whether oxytocin affects the acquisition or the consolidation of intrusive memories. The findings indicate that in healthy participants oxytocin influences subsequent intrusive memories when administered during acquisition, but not during the consolidation of a trauma analogue.

Contradictory to our findings, the administration of oxytocin directly after exposure to stress did show anxiolytic effects and decreased PTSD symptoms in PTSD patients [40, 43]. A possible hypothesis for the missing effect of oxytocin on the consolidation of intrusive memories in our study might be the degree of distress caused by the trauma film paradigm. The results of van Zuiden et al. [40] indicate that oxytocin does not generally decrease PTSD symptoms, but is rather only effective in individuals that reported high levels of distress after the trauma. It seems plausible that the trauma film paradigm presented to healthy subjects does not have the same impact as actual traumatic events and does not cause the same level of distress. Oxytocin might therefore not significantly reduce symptoms when applied in trauma analogue studies. Further Scheele et al. [69] found the effect of oxytocin to be dependent on trauma disclosure in PTSD patients. Suggesting that trauma disclosure facilitates social support, and the effect of social support, in turn, is mediated by oxytocin. A second hypothesis for the lack of effect of oxytocin on the consolidation of intrusive memories is the administration of a single dose of nasal oxytocin only. Both Scheele et al. [69] and van Zuiden et al. [40] administered nasal oxytocin several times during the consolidation phase. A repeated administration may have altered the results of the current study.

### Neurobiological and genetic factors influencing the number of intrusive memories

We replicated previous findings [12], showing an association between the PTSD PRS on the number of intrusive memories independent of treatment condition. This is in line with studies showing an increased likelihood of developing PTSD in individuals with higher polygenic loadings for PTSD [13, 14, 70, 71]. As intrusions are a hallmark symptom of PTSD, it is plausible that genetic vulnerability for PTSD affects the occurrence of intrusive memories. It is remarkable to repeatedly find this association applying a trauma analogue, although this is a relatively mild stressor [72] and the sample consisted of healthy participants only. That finding strongly suggests an influence of PTSD PRS on the development of key PTSD symptoms.

Further, a stronger cortisol and sAA response to the film paradigm was positively associated with intrusive memories suggesting that a heightened biological stress response is predictive of intrusive memories after a trauma analogue. This finding is consistent with previous studies showing a positive association between increased salivary cortisol levels and elevated noradrenergic activation and intrusive memories [9, 11, 73, 74].

### Psychological factors influencing the number of intrusive memories

Poisson regression revealed associations between the emotion regulation strategy cognitive reappraisal and trait anxiety and intrusive memories. Both constructs have been associated with intrusions before [48]. Individuals using cognitive reappraisal, aim at regulating the emotional impact of a stimulus by reinterpreting its meaning [75]. Cognitive reappraisal has been identified as a resilience factor to an adverse event [76–79] and for PTSD [70, 80]. Accordingly, we found a negative association between the habitual use of the emotion regulation strategy cognitive reappraisal and the number of intrusive memories. A possible neurobiological explanation for this association is reduced activation of the amygdala during emotional processing and increased activation of the prefrontal cortex [78, 81], resulting in a neural functioning that favors a successful regulation of negative emotions [82] and prevents intrusions. The current study extends prior research showing a negative association between the habitual use of cognitive reappraisal and intrusive memories. Previous studies have also shown fewer intrusive memories after cognitive reappraisal training [83]. Future research should therefore target cognitive reappraisal training as a possible preventive strategy for PTSD in populations at risk. With regard to pre-trauma trait anxiety, our results confirm previous publications showing a positive association with PTSD and therefore identify high trait anxiety as a vulnerability factor for PTSD [48, 84]. Impaired processing of the trauma film in individuals with high trait anxiety seems likely, as high trait anxiety is associated with self-regulatory executive dysfunctions and emotion regulation deficits [84–86]. Furthermore, studies revealed molecular alterations in the brain in individuals with high trait anxiety, resulting in an increased vulnerability to stress [87].

### Strength and limitations

The high internal validity of this double-blinded randomized placebo-controlled study due to high experimental control, strict inclusion criteria, inclusion of women only, and the clear operationalization of intrusive memories can be highlighted as the main strength. Menstrual cycle [88] and intake of hormonal contraceptives [89] have an impact on endogenous oxytocin concentrations and intrusion formation [57, 90]. Therefore, we controlled for the intake of hormonal contraceptives and tested naturally cycling women only during their luteal cycle phase.

Some limitations to the study should be named. Oxytocin has sexually dimorphic effects that may be due to sex-specific differences in oxytocin plasma concentrations [91] or different oxytocin expressions in different brain regions of women and men [92]. Therefore, only female participants were included in the study, and a trauma film, which was tailored to female participants [65] was applied. While this can be considered a strength in terms of internal validity it hampers generalization to men. Testing oxytocin effects in men may reveal different results. Further, inclusion criteria were broadened, compared to the previously published study [12] (age 18 to 40 vs. 18 to 30; women with all levels of education were included vs. only students). Nevertheless, the sample still consisted of healthy young women without any previous traumatic experiences. Although female sex and young age depict risk factors for PTSD [5], the current results cannot be generalized to men and more vulnerable populations, with a psychiatric diagnosis or previous traumatic experiences. Results need to be extended to a more heterogeneous sample. The relatively mild stressor is a further limitation of the study, as it differs from real traumatic events causing PTSD. It is not clear if conclusions about the development of intrusions in patients with PTSD can be drawn with the presented data. Furthermore, self-report measures of intrusions create the risk of response bias [93]. Nevertheless, trauma film paradigms offer an ethically justifiable and valid investigation of pre- and peri-traumatic PTSD risk factors. They also offer a valuable method to investigate pre-existing vulnerabilities that put some individuals at a higher risk to develop intrusive memories [72]. Further, according to Holmes and Bourne [94], we defined intrusions by frequency, modality, content, liveliness, and degree of distress as these features of intrusions have been shown to predict a PTSD diagnosis [22, 25, 26, 95].

## Conclusion

Exogenous administration of oxytocin during the consolidation of trauma did not affect the formation of intrusive memories in the present study. Combining the current results with a previously conducted study [12], it appears that oxytocin influences the acquisition but not the consolidation of intrusive memories in healthy young females after an analogue trauma. Variables that were related to the development of intrusions were PTSD PRS, HPA axis and noradrenergic response to the film, high trait anxiety, and the emotion regulation strategy reappraisal. These findings contribute to a better understanding of the development of PTSD and should be further investigated as they can potentially be targeted to prevent PTSD after a traumatic event.

## Supplementary information

Supplement

Supplemental Figure 1

Supplemental Figure 2

Supplemental Figure 3

Supplemental Figure 4

Supplemental Figure 5
