# Supplementary material for: Oxytocin vs. placebo effects on intrusive memory consolidation using a trauma film paradigm: a randomized, controlled experimental study in healthy women
Source: Transl Psychiatry. 2023 Feb 4;13:42. doi: 10.1038/s41398-023-02339-z (PMC9899212; doi:10.1038/s41398-023-02339-z)
Supplement: Supplementary file 1 — Supplement [file 41398_2023_2339_MOESM1_ESM.docx]

**Oxytocin vs. placebo effects on intrusive memory consolidation using a trauma film paradigm: a randomized, controlled experimental study in healthy women**

**Supplement**

**Methods**

**Extended Methods and Materials**

***Participants***

Exclusion criteria contained: (1) history of trauma, (2) any lifetime or current Axis-I disorder, as screened for by the German version of the Structured Clinical Interview for DSM-IV (SCID-I) [1], (3) blood phobia (because of the depiction of violence and bleeding injuries in the trauma film), (4) any physical illness, (5) any medication intake (except hormonal contraceptives), (6) a Body Mass Index (BMI) > 30, (7) pregnancy or lactation, (8) excessive sport (e.g. training for a marathon), (9) excessive nicotine consumption (more than one package a day).

**Psychometric assessment**

Following questionnaires were used to root out psychopathology in participants and to consider possible influences on the formation of intrusive memories: (1) Beck Depression Inventory-Revised (BDI-II) [2], to detect potential depressive symptoms. A BDI-II score > 13 implies clinically relevant depressive symptoms [2] and led to exclusion, (2) Childhood Trauma Questionnaire [3], to detect any childhood trauma that possibly impact the noradrenergic responses to stress [4] and can increase the risk of post-traumatic stress disorder (PTSD) after a secondary trauma in adulthood [5], (3) Emotion Regulation Questionnaire [6], to identify emotion regulation strategies, as these can affect the formation of intrusions [7], (4) State-Trait Anxiety Inventory [8], to determine differences in anxiety, as pre- trauma trait anxiety is positively associated with PTSD [9].

***Trauma film***

Participants watched a well-established trauma film paradigm [10-12], which reliably evokes intrusive symptoms [12-14], in a dark room on a 2x2.5m screen. The volume of the sound was standardized and played via headphones. The film scene shows a section of the commercial film *Irreversible* by Gaspar Noë [15]. The section (14 minutes, 40 seconds) displays very detailly how a woman is attacked, brutally raped and beaten up by a stranger in a pedestrian underpass. Before watching the film, participants were asked not to close their eyes and to allow emerging feelings. During the film, a female experimenter was present to verify that the participants watched it attentively and did not take off the headphones.

***Intrusion diary***

Intrusive memories were measured with a paper-pencil intrusion diary, which has been used in previous studies [13, 16], for four days subsequent to the trauma film. Participants were asked to enter every memory of the film directly after occurrence. To ensure participation and prevent data loss, participants were asked to transfer their records into an online diary every evening and received a message at 9 pm every day as a reminder. According to Holmes et al. [11] the diary included information about the memory content, modality, vividness and degree of distress (0 = not at all to 5 = very strong). Further, the degree of distress and the vividness of every memory were rated on a scale from 0 (*not at all*) to 5 (*a lot*). Memories were classified as intrusive if they occurred spontaneously, included images and had ratings > 0 of the degree of distress and vividness [17, 18].

***Salivary assessment***

Saliva samples were collected in salivettes (blue cap, Sarstedt, Germany), in order to measure free cortisol and sAA. Samples were immediately stored at – 80 °C (-112 °F) until biochemical analysis. Biochemical analysis were conducted in the Neurobiology Laboratory of the Department of Psychiatry and Neuroscience, Charité – Universitätsmedizin Berlin. Detailed description of the analyses have been published previously [19]. We calculated delta values to indicate increases in salivary cortisol and sAA activity, by subtracting the baseline measurement from the maximum measurement between + 60 and + 75 min.

***Heart rate variability***

A heart rate monitor and a Polar Electro Oy chest strap (Polar RS800CX; Kempele, Finland) have been used to measure Heart rate variability (HRV). This economic method has been shown to be a valid and reliable alternative to an electrocardiogram [20]. HRV was measured for five minutes, while participants were asked to remain seated in a relaxed position with their feet on the floor and hands on their lap in a quiet room. The female examiner was present at all times to assure adherence to instructions. The mean values of the previous and the following inter-beat intervals were calculated and used as interpolated intervals to replace abnormal intervals, in order to detect and replace artifacts. Filter power was set as “moderate” at default and the minimum protection zone was set at six beats/minute (BPM). Kubios HRV Version 2.0 (Kubios Oy, Kuopio, Finland) for MATLAB Runtime MCR (R2016b) was utilized to analyze data. The root mean square of successive differences (RMSSD) was used as the HRV parameter for statistical analysis. The RMSSD has been preferred to low-frequency power and low-frequency/high-frequency ratios, as it is relatively free of sympathetic inference and respiration rate and therefore a valid indicator of primarily parasympathetic nervous system activity, when measured at rest [21].

**Supplemental Table 1.** Number of SNPs comprised in each PRS

| **PRS** | **No. of SNPs** |
| --- | --- |
| Cross disorder | 111,381 |
| MDD | 107,087 |
| PTSD | 161,809 |
| PTSD female | 161,519 |
| SCZ | 102,573 |

**Note**. PRS = Polygenic Risk Score, SNP = single nucleotide polymorphism, MDD = Major Depression Disorder, PTSD = Post-traumatic Stress Disorder, SCZ = Schizophrenia

| **Characteristics** | **Included (*n* = 202)**  *M* (*SD*) or *n* | **Excluded (*n* = 15)**  *M* (*SD*) or *n* | **Statistics** |
| --- | --- | --- | --- |
| Age | 25.13 (5.99) | 25.47 (5.87) | *t*(215) = 0.21 *p* =0 .84, *d* = -0.06 (95 % CI [-2.82, 3.49]) |
| Intake of oral contraceptives | 59 (29.21 %) | 2 (13.33 %) | $\chi$^2^*(*1) = 1.74, *p* = 0.19, *φ* = 0.09 |
| Current smoker | 55 (27.22 %) | 5 (33.33 %) | $\chi$*^2^*(1) = 0.26, *p* = 0.61, *φ* = -0.04 |
| BMI | 22.02 (2.65) | 22.54 (3.08) | *t*(215) = 0.73, *p* = 0.47, *d* = -0.18 (95 % CI [-.89, 1.93]) |
| CTQ | 32.02 (8.32) | 38.00 (11.95) | *U* = 1086.5, Z = -1.83 *p* = 0.07 |
| STAI-T | 32.59 (6.58) | 35.14 (10.79) | *U* = 1307.00, *Z* = -0.42, *p* = 0.68 |
| BDI-II | 4.16 (3.84) | 7.53 (6.94) | U = 1074.00, Z = -1.89, p = 0.06 |
| ERQ reappraisal | 29.47 (5.39) | 28.27 (7.65) | *t*(215) = -0.81, *p* = 0.42, *d* = 0.18 (95 % CI [-4.14, 1.73]) |
| ERQ suppression | 11.84 (4.34) | 11.40 (4.98) | *t*(215) = -38, *p* = 0.71, *d* = 0.09 (95 % CI [-2.76, 1.87]) |
| Participants who had seen the film before | 14 (6.93 %) | 0 (0.00 %) | $\chi$*^2^*(1) = 0.82, *p* = 0.37, *φ* = -0.06 |

**Supplemental Table 2** Included vs. Excluded Participants

**Note:** *M* = mean, *SD* = standard deviation, BMI = body mass index, CTQ = Childhood Trauma Questionnaire, STAI-T = state-trait anxiety inventory-trait subscale, BDI-II = Beck depression inventory-revised, ERQ = emotion regulation questionnaire (subscales reappraisal and suppression). In case the normality assumption for residuals was violated, the Mann–Whitney *U* test was used (*U*).

**References**

1. Wittchen, H.-U., M. Zaudig, and T. Fydrich, *Skid. Strukturiertes klinisches Interview für DSM-IV. Achse I und II. Handanweisung.* 1997.

2. Beck, A.T., R.A. Steer, and G.K. Brown, *Manual for the beck depression inventory-II.* San Antonio, TX: Psychological Corporation, 1996. **1**: p. 82.

3. Wingenfeld, K., C. Spitzer, C. Mensebach, H.J. Grabe, A. Hill, U. Gast, et al., *The German version of the Childhood Trauma Questionnaire (CTQ): preliminary psychometric properties.* Psychotherapie, Psychosomatik, Medizinische Psychologie, 2010. **60**(11): p. 442-450.

4. Otte, C., T.C. Neylan, N. Pole, T. Metzler, S. Best, C. Henn-Haase, et al., *Association between childhood trauma and catecholamine response to psychological stress in police academy recruits.* Biological psychiatry, 2005. **57**(1): p. 27-32.

5. Breslau, N., H.D. Chilcoat, R.C. Kessler, and G.C. Davis, *Previous exposure to trauma and PTSD effects of subsequent trauma: results from the Detroit Area Survey of Trauma.* American journal of Psychiatry, 1999. **156**(6): p. 902-907.

6. Abler, B. and H. Kessler, *Emotion regulation questionnaire–Eine deutschsprachige Fassung des ERQ von Gross und John.* Diagnostica, 2009. **55**(3): p. 144-152.

7. Kaczkurkin, A.N., Y. Zang, N.G. Gay, A.L. Peterson, J.S. Yarvis, E.V. Borah, et al., *Cognitive emotion regulation strategies associated with the DSM‐5 posttraumatic stress disorder criteria.* Journal of Traumatic Stress, 2017. **30**(4): p. 343-350.

8. Laux, L., *Das State-Trait-Angstinventar (STAI): theoretische grundlagen und handanweisung.* 1981.

9. McNally, R.J., J.P. Hatch, E.M. Cedillos, C.A. Luethcke, M.T. Baker, A.L. Peterson, et al., *Does the repressor coping style predict lower posttraumatic stress symptoms?* Military medicine, 2011. **176**(7): p. 752-756.

10. Holmes, E.A. and C. Bourne, *Inducing and modulating intrusive emotional memories: A review of the trauma film paradigm.* Acta psychologica, 2008. **127**(3): p. 553-566.

11. Holmes, E.A., C.R. Brewin, and R.G. Hennessy, *Trauma films, information processing, and intrusive memory development.* Journal of Experimental Psychology: General, 2004. **133**(1): p. 3.

12. Weidmann, A., A. Conradi, K. Gröger, L. Fehm, and T. Fydrich, *Using stressful films to analyze risk factors for PTSD in analogue experimental studies–which film works best?* Anxiety, Stress, & Coping, 2009. **22**(5): p. 549-569.

13. Schultebraucks, K., T. Maslahati, K. Wingenfeld, J. Hellmann-Regen, J. Kraft, M. Kownatzki, et al., *Intranasal oxytocin administration impacts the acquisition and consolidation of trauma-associated memories: a double-blind randomized placebo-controlled experimental study in healthy women.* Neuropsychopharmacology, 2021: p. 1-9.

14. Rombold, F., K. Wingenfeld, B. Renneberg, J. Hellmann-Regen, C. Otte, and S. Roepke, *Influence of the noradrenergic system on the formation of intrusive memories in women: An experimental approach with a trauma film paradigm.* 2016.

15. G Noé, T.B., M Bellucci, V Cassel,A Dupontel *Irreversible*. 2002: France.

16. Rombold, F., K. Wingenfeld, B. Renneberg, F. Schwarzkopf, J. Hellmann-Regen, C. Otte, et al., *Impact of exogenous cortisol on the formation of intrusive memories in healthy women.* Journal of psychiatric research, 2016. **83**: p. 71-78.

17. Ehlers, A., A. Hackmann, and T. Michael, *Intrusive re‐experiencing in post‐traumatic stress disorder: Phenomenology, theory, and therapy.* Memory, 2004. **12**(4): p. 403-415.

18. Arntz, A., C. de Groot, and M. Kindt, *Emotional memory is perceptual.* Journal of Behavior Therapy and Experimental Psychiatry, 2005. **36**(1): p. 19-34.

19. Schultebraucks, K., F. Rombold-Bruehl, K. Wingenfeld, J. Hellmann-Regen, C. Otte, and S. Roepke, *Heightened biological stress response during exposure to a trauma film predicts an increase in intrusive memories.* Journal of abnormal psychology, 2019. **128**(7): p. 645.

20. Laborde, S., E. Mosley, and J.F. Thayer, *Heart Rate Variability and Cardiac Vagal Tone in Psychophysiological Research - Recommendations for Experiment Planning, Data Analysis, and Data Reporting.* Front Psychol, 2017. **8**: p. 213.

21. Laborde, S., E. Mosley, and J.F. Thayer, *Heart rate variability and cardiac vagal tone in psychophysiological research–recommendations for experiment planning, data analysis, and data reporting.* Frontiers in psychology, 2017. **8**: p. 213.

**Supplemental Figure 1.** Chronological overview of the study procedure.

**Supplemental Figure 2.** Scatterplot mapping individuals based on the first two principal components (PC) obtained from genotype principal component analysis. Cut-off values for PC1 (> 0.05) and PC2 (< -0.05) were selected to exclude individuals of non-European ancestry.

**Supplemental Figure 3**. Physiological stress response in the course of the study – salivary α- amylase activity (top left), heart rate variability measured as root mean square of successive differences (RMSSD) (top right) and salivary cortisol
Note. Points are means, with standard errors represented by vertical bars.

**Supplemental Figure 4.** Overlap assumption was fulfilled. The estimated propensity scores were not close to 0 or 1.

**Supplemental Figure 5.** Variable importance ranking of the covariates that are best at predicting differences in the effect of oxytocin on intrusive memories after exposure to a trauma film.

CTQ = childhood trauma questionnaire, Schizophrenia PRS = Schizophrenia polygenic risk score, delta sAA = salivary α- amylase, ERQ - R = emotion regulation questionnaire – reappraisal, STAI trait = state trait anxiety inventory – subscale trait, cross PRS = cross – disorder polygenic risk score, MDD PRS = major depressive disorder polygenic risk score, PTSD PRS = post-traumatic stress disorder polygenic risk score, ERQ – S = emotion regulation questionnaire suppression.
