# Supplementary material for: Oxytocin vs. placebo effects on intrusive memory consolidation using a trauma film paradigm: a randomized, controlled experimental study in healthy women
Source: Transl Psychiatry. 2023 Feb 4;13:42. doi: 10.1038/s41398-023-02339-z (PMC9899212; doi:10.1038/s41398-023-02339-z)
Supplement: Supplementary file 2 — Supplemental Figure 1 [file 41398_2023_2339_MOESM2_ESM.pdf]

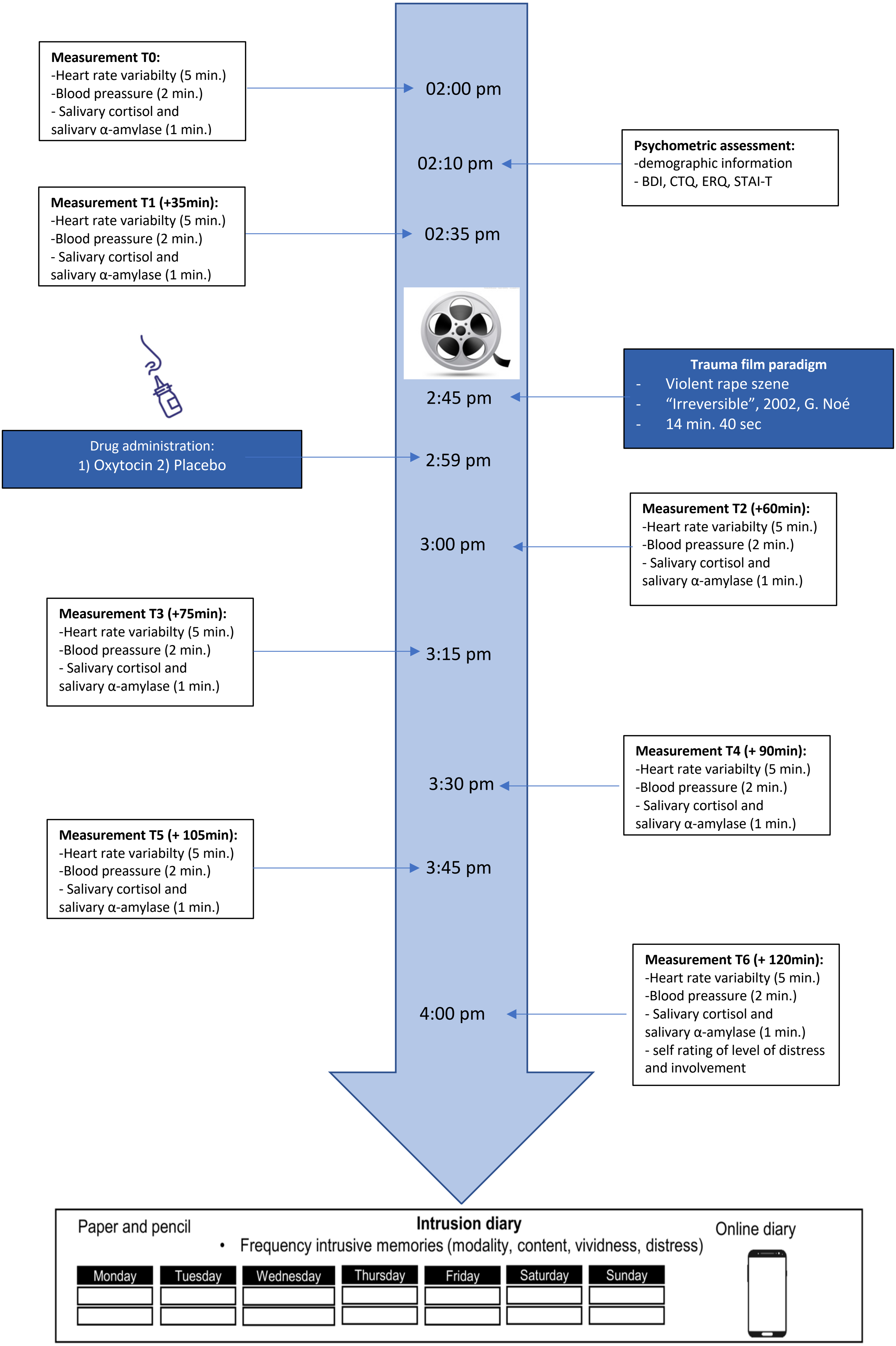
Measurement T0:
-Heart rate variabilty (5 min.)
-Blood preassure (2 min.)
- Salivary cortisol and
salivary α-amylase (1 min.)
02:00 pm
Psychometric assessment:
-demographic information
- BDI, CTQ, ERQ, STAI-T
02:10 pm
Measurement T1 (+35min):
-Heart rate variabilty (5 min.)
-Blood preassure (2 min.)
- Salivary cortisol and
salivary α-amylase (1 min.)
02:35 pm
Trauma film paradigm
- Violent rape szene
- “Irreversible”, 2002, G. Noé
- 14 min. 40 sec
2:45 pm
Drug administration:
1) Oxytocin 2) Placebo
2:59 pm
Measurement T2 (+60min):
-Heart rate variabilty (5 min.)
-Blood preassure (2 min.)
- Salivary cortisol and
salivary α-amylase (1 min.)
3:00 pm
Measurement T3 (+75min):
-Heart rate variabilty (5 min.)
-Blood preassure (2 min.)
- Salivary cortisol and
salivary α-amylase (1 min.)
3:15 pm
Measurement T4 (+ 90min):
-Heart rate variabilty (5 min.)
-Blood preassure (2 min.)
- Salivary cortisol and
salivary α-amylase (1 min.)
3:30 pm
Measurement T5 (+ 105min):
-Heart rate variabilty (5 min.)
-Blood preassure (2 min.)
- Salivary cortisol and
salivary α-amylase (1 min.)
3:45 pm
Measurement T6 (+ 120min):
-Heart rate variabilty (5 min.)
-Blood preassure (2 min.)
- Salivary cortisol and
salivary α-amylase (1 min.)
- self rating of level of distress
and involvement
4:00 pm
Paper and pencil
Intrusion diary
• Frequency intrusive memories (modality, content, vividness, distress)
Online diary
Monday
Tuesday
Wednesday
Thursday
Friday
Saturday
Sunday
