# Supplementary figures and images for: Oxytocin vs. placebo effects on intrusive memory consolidation using a trauma film paradigm: a randomized, controlled experimental study in healthy women
Source: Transl Psychiatry. 2023 Feb 4;13:42. doi: 10.1038/s41398-023-02339-z (PMC9899212; doi:10.1038/s41398-023-02339-z)

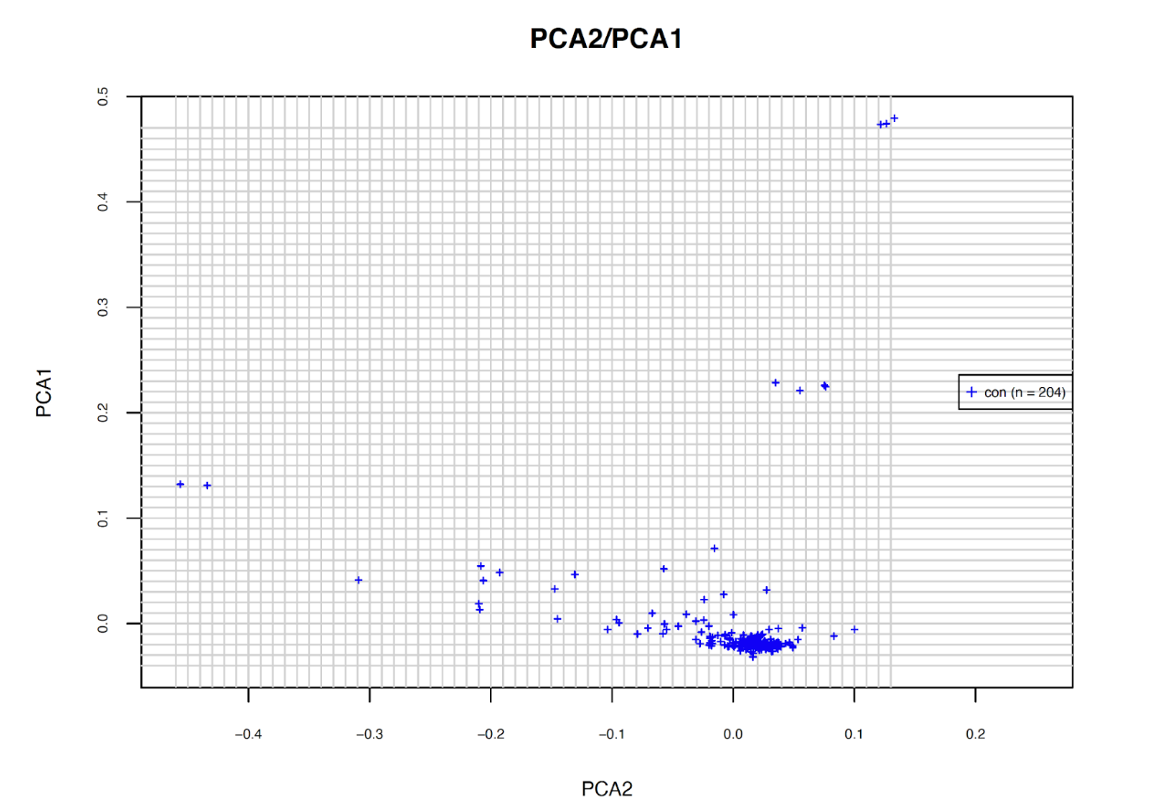

Supplement: Supplementary file 3 — Supplemental Figure 2 [file 41398_2023_2339_MOESM3_ESM.png]

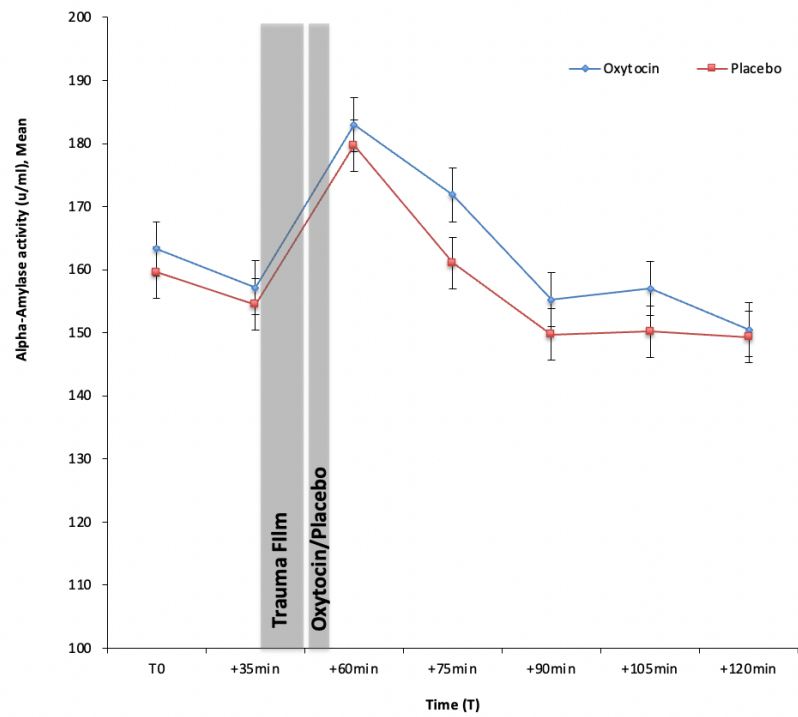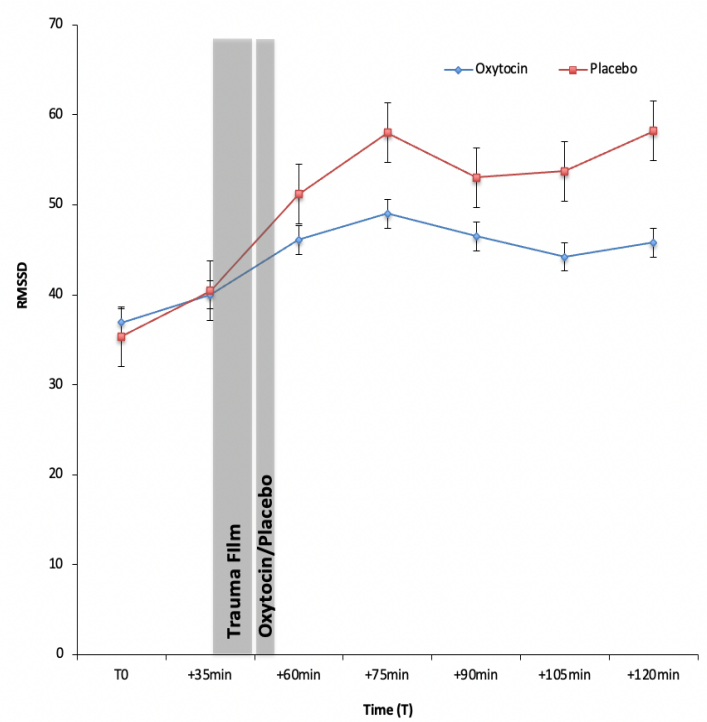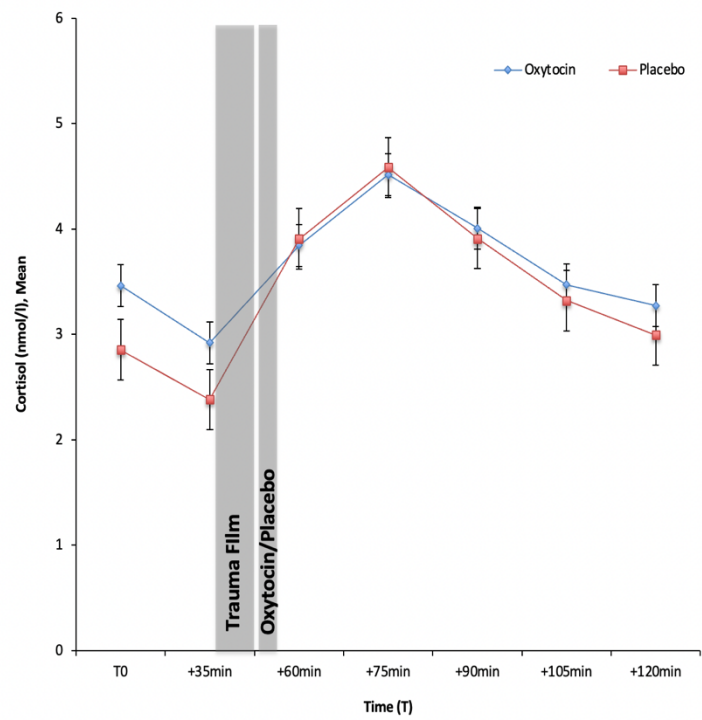

Supplement: Supplementary file 4 — Supplemental Figure 3 [file 41398_2023_2339_MOESM4_ESM.pdf]

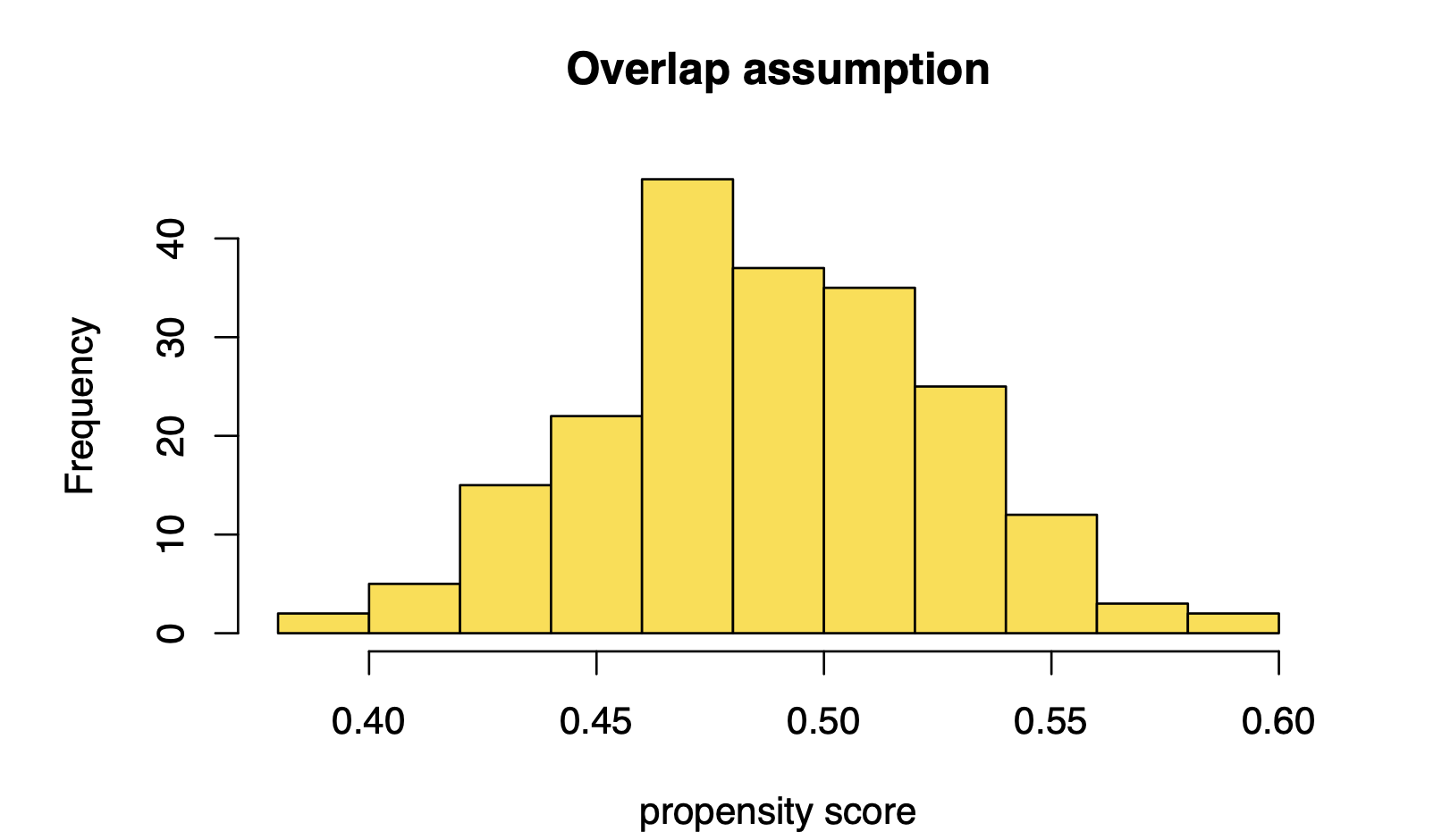

Supplement: Supplementary file 5 — Supplemental Figure 4 [file 41398_2023_2339_MOESM5_ESM.png]

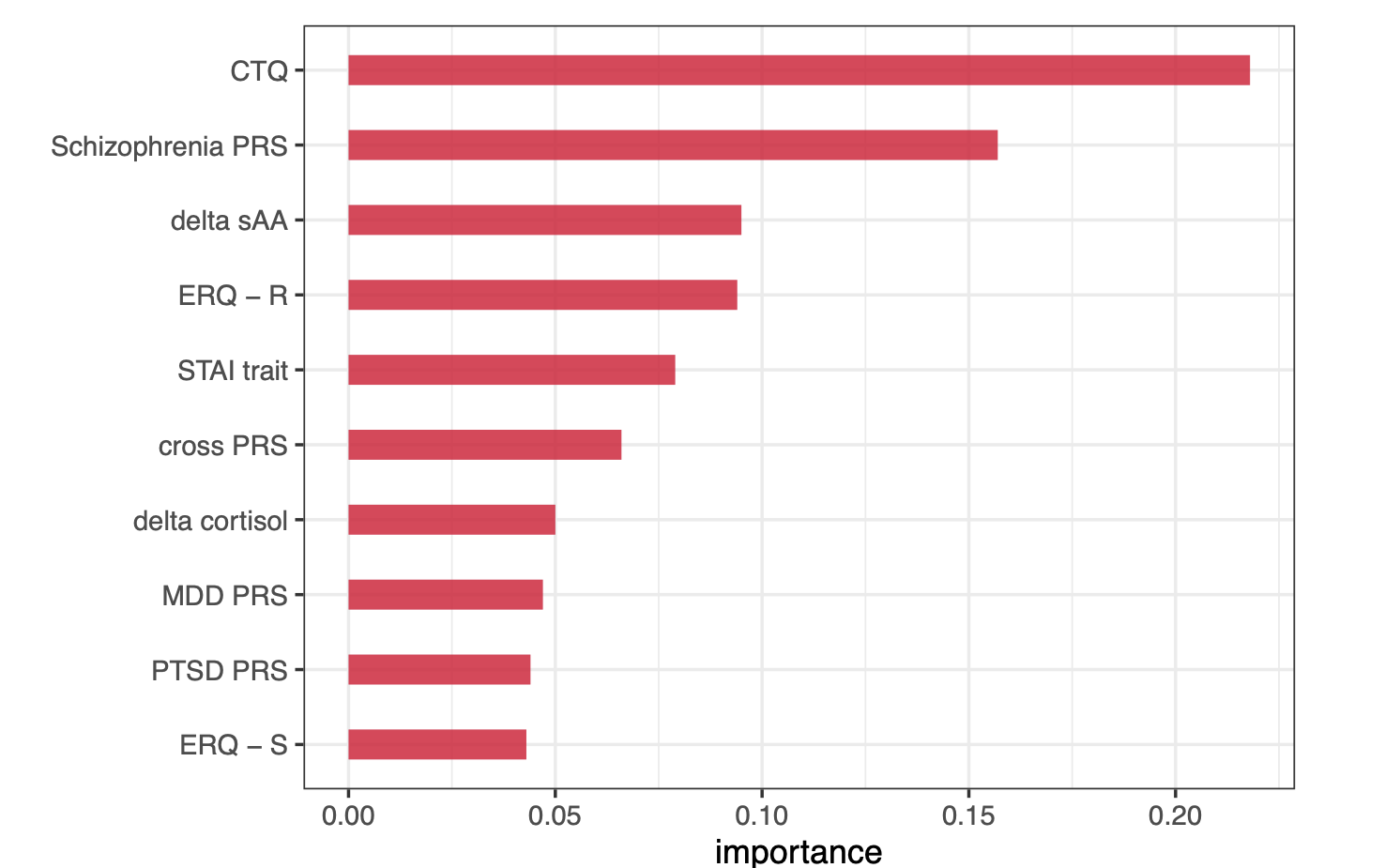

Supplement: Supplementary file 6 — Supplemental Figure 5 [file 41398_2023_2339_MOESM6_ESM.png]
